# Adapting Adolescent Dating Violence Prevention Interventions to Victims of Child Sexual Abuse

**DOI:** 10.1177/15248399221083255

**Published:** 2022-09-08

**Authors:** Geneviève Brodeur, Mylène Fernet, Martine Hébert, Christine Wekerle

**Affiliations:** 1 Université du Québec à Montréal, Montreal, Québec, Canada; 2 McMaster University, Hamilton, Ontario, Canada

**Keywords:** teen dating violence, revictimization, context adaptation of intervention, adolescent violence prevention

## Abstract

Considering the increased risk of revictimization, adolescents who have experienced child sexual abuse (CSA) are a priority subpopulation for the prevention of dating violence. Yet, intervention programs often focus on psychological symptomology associated with CSA; few tackle issues specific to relational violence. Addressing the relational traumatization of adolescents with a history of CSA is essential to prevent their revictimization. Given specific CSA sequelae related to intimacy and engagement in sexual behaviors, there is a need for tailoring interventions to boy and girl survivors. A case study of a group intervention designed for adolescent girls with a history of CSA was conducted. The context adaptation, based on intervention mapping proposed by Bartholomew and colleagues, served as a theoretical framework. Four steps were taken to ensure that the intervention addressed CSA youth needs: (a) needs assessment, (b) analysis of the conceptual framework of the original program, (c) selection of interventions and developing new interventions, and (d) validation with a committee of practitioners. This approach provided an understanding of risk factors and intervention priorities using the problem logic model. The original program was enhanced by adding four interventions addressing the prevention of dating violence. These interventions were then validated by practitioners before implementation in the setting. The approach underscores the relevance of understanding the needs of the clientele and of adopting a collaborative approach to ensure the proposed interventions are relevant.

## Background

Global prevalence rates of child sexual abuse (CSA) suggest that 8% to 31% of girls and 3% to 17% of boys report having been victims before the age of 18 (Barth et al., 2013). Adolescent dating violence (ADV) is a serious issue affecting all youth, but some groups more than others. According to the 2017 U.S. Youth Risk Behavior Survey, among youth with current/past year dating (68.3%), 6.4% of heterosexual youth reported receiving physical dating violence, while gay/bisexual youth (17.2%) and youth unsure of their sexual identity (14.1%) reported the highest rates. In a Canadian high school population study, about one-third reported psychological ADV; about 15% reported physical and/or sexual ADV. Findings revealed that both girl and boy victims of CSA reported higher rates of psychological, physical, and sexual dating violence compared to non-CSA adolescents (Hébert et al., 2017).

Victims of CSA suffer various negative consequences that can affect daily functioning, interfere with their interpersonal relationships, and persist into adulthood (Afifi et al., 2016; Amédée et al., 2019). One of the particularly worrying consequences of CSA is the increased risk of revictimization and sexual risk-taking, including later intimate partner violence (Scoglio et al., 2019; Wekerle et al., 2017). Indeed, several studies have shown that girls with a history of CSA are more likely to sustain psychological, physical, and sexual victimization in the context of their first romantic relationships (Hébert et al., 2017; Walker et al., 2019). Boys and girls receiving child welfare services in adolescence with a self-reported history of CSA rated their motives for having sexual activities strongly in terms of coping, which mediated the CSA-sexual risk-taking relationship (Wekerle et al., 2017). Preventing revictimization in this vulnerable clientele is thus of utmost importance. The phenomenon of revictimization in romantic relationships among adolescent victims of CSA and the scientific literature associated with the best practices to prevent revictimization remains fragmented.

Following disclosure of CSA, victims may benefit from interventions ranging from individual, family, or group therapies (Brown et al., 2020). Group intervention is one of the most frequent intervention modalities for adolescents (Caro et al., 2019), and may be viewed as a cost-effective strategy that offers participants the opportunity to share difficult experiences with peers in a secure setting (Burlingame et al., 2018). Despite the fact that ADV is a major issue among CSA victims, the majority of group interventions tackle consequences associated with CSA (anxiety, depression, and feelings of guilt), often neglecting to foster the development of healthy romantic relationships and the prevention of dating violence (Hébert et al., 2012).

### Promising Venue to Decrease Revictimization Among CSA Adolescent Clienteles

Generally, ADV prevention programs are offered in school settings and opt for a universal approach by addressing the general population, without adapting interventions to the specific needs of at-risk groups (Levesque et al., 2016). Yet there is some evidence that suggests that universal programs might not be as effective for CSA victims (Crooks et al., 2019). This underscores the need to also implement targeted prevention efforts. To tailor interventions to the needs of adolescent girls who experienced CSA, we must consider the increased risk of revictimization and address dating relationships issues (Hébert et al., 2021), such as difficulties in recognizing abusive behaviors and ending an abusive relationship (Castro & Santos-Iglesias, 2016). By ensuring such targeted intervention-based needs assessment of the population, the risk factors that contribute to revictimization must be considered (Crooks et al., 2019).

It is crucial to understand the risk factors that render this clientele more vulnerable to ADV (Boivin et al., 2014). To do so, Lau and Kristensen (2010) suggest developing, implementing, and evaluating interventions that target this increased vulnerability by focusing on risk factors, including symptoms of psychological distress, inadequate emotional regulation, and difficulties recognizing ADV. Bartholomew and colleagues (2016) developed a systematic and iterative method, Intervention Mapping (IM), to adapt public health interventions to specific contexts. This process aims to modify an intervention while maintaining its fundamental characteristics (e.g., the modalities of intervention) and the conceptual model of the original version (e.g., the essential elements). Context adaptation increases the relevance of the original intervention by making the intervention more understandable and adapted to the new clientele group. In the victimization field, such an approach has been successfully used to adapt the *Families for Safe Dates* program, a universal dating violence prevention program to youth exposed to domestic violence, a high-risk clientele (Foshee et al., 2015).

#### Aims

Considering the necessity to target specific needs of adolescents who have experienced CSA to prevent revictimization, this article describes the context adaptation of interventions to enhance a program designed for teen victims of CSA. More precisely, this article aims to (a) analyze the conceptual framework of the program offered; (b) adapt and develop interventions to prevent revictimization; and (c) validate these interventions through a collaborative process.

## Method

A case study approach was used to supplement an existing group program for CSA youth victims by adding specific interventions that address specially the risk of ADV. The setting selected for this case study is a community organization offering a group intervention for teen victims of CSA in the province of Quebec, Canada. The group intervention is based on a psychoeducational approach. The group aims to help teenagers regain power and balance in their lives through, among other things, discussions, individual work, and role-playing.

The method followed was based on IM (Bartholomew et al., 2016). The first four steps of the method, which relate more specifically to the adaptation and development of an intervention, were used with some adaptations. This four steps process is detailed in Table 1. Practitioners, which are project stakeholder, collaborated with the research team and provided input on priority needs for youth, shared the original program materials, and offered feedback on the planned interventions produced that was then applied.

**Table 1:** Summary of Steps Taken and Associated Tasks

Steps	Tasks
1. Needs assessment	1.1 Conduct a needs assessment by reviewing the literature and consultation with a key informant that works with the clientele 1.2 Identify the personal, behavioral, and environmental determinants that are the causes of the problem 1.3 Creating a logic model of the problem (Figure 1) 1.4 Identification of the behavioral and environmental objectives following the analysis of the problem 1.5 Creating a matrix of change (Table 2)
2. Conceptual framework of the original program	2.1 Thematic analysis of the original program 2.2 Creation of a logic model of the original program (Table 3) 2.3 Identification of what is addressed and not addressed in the matrix in order to improve the original program (i.e., learning objectives and risk factors) 2.4 Taking the decision to adapt and create new interventions to improve the original program.
3. Selection of interventions and development of new interventions	3.1 Thematic analysis of potential interventions to adapt to 3.2 Selection of interventions to adapt to according to their relevance to the clientele and the matrix objectives 3.3 Context adaptation on the selected interventions: (a) include or remove content that would be irrelevant, (b) emphasize content deemed to be important, and (c) addition of content deemed important discussed with the public 3.4 Creation of new interventions (see Table 4 for an overview of the interventions) 3.5 Ensure that there is enough practical application to meet all the objectives of the matrix 3.6 Consultation with a researcher that has experience in program creation and dating violence prevention
4. Validation with a committee of practitioners	4.1 Focus group with practitioners (stakeholders) 4.2 Thematic analysis of the focus group 4.3 Apply suggested changes to the interventions

### Step 1: Needs Assessment

Needs assessment aims to understand health problems, based on a socio-ecological model (Bartholomew et al., 2016). To do so, a literature review was conducted to document personal, behavioral, and environmental determinants (e.g., risk factors) helping to explain the problem under investigation. In addition, a practitioner who leads the intervention groups for adolescent girls was invited to respond to questions about the needs of the clientele. Examples of questions include: “What would be relevant topics to discuss with participants in the group, regarding sexuality and relationships?,” “Do they have any dissatisfaction with any interventions (e.g., content, practical application) currently in the actual program?” and “Are there any interventions that are particularly popular and why?.”

**Figure 1:**
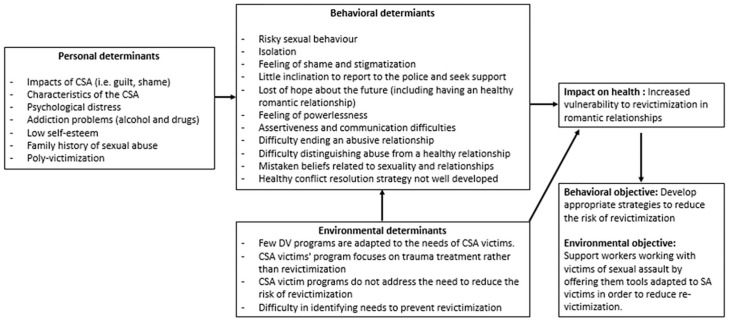
Logic Model of the Problem

At this step, a logic model of the problem (Figure 1), which summarized the risk factors of revictimization and thus, helped identify the intervention priorities, was developed. Once the ultimate objective (or behavioral objective) of the program was identified, a matrix of change objectives was proposed. This matrix brings together performance objectives and learning objectives that are formulated based on the personal and external determinants identified in the matrix (see Table 2 for a matrix summary).

**Table 2:** Examples of Performance Objectives and Learning Objectives

Performance objectives						
	During the course of the interventions, youth develop skills to . . .					
	Become aware of the importance of oneself and living in healthy and egalitarian relationships.	Express their intolerance of all types of violence.	Recognize the increased vulnerability of revictimization for adolescent girls who have experienced CSA.	Recognize that CSA leaves behind erroneous beliefs about love and sexual relationships.	Apply appropriate interpersonal strategies to prevent ADV.	Get help as soon as they feel the need.
Personal determinants						
	Learning objectives		Girls learns to . . .			
Knowledge	Describe the components of a healthy, egalitarian romantic relationship.	Recognize the different types of ADV.	Understand the prevalence of DV in CSA victims.	Identify erroneous beliefs about love and sexual relationships.	Know the principles of effective and appropriate communication in their interpersonal relationships.	Designate trustworthy and accessible people as resource persons.
Attitudes	Define their conception of a healthy and egalitarian romantic relationship.	Verbalize their beliefs about violence in relationships.	Recognize the importance of working on their erroneous beliefs about love and sex.	Recognize the importance of using effective and appropriate communication principles in their interpersonal relationships.	Recognize that seeking help makes a difference.	
Abilities	Recognize the components of a healthy, egalitarian romantic relationship.	Feeling able to identify different types of violence in a romantic relationship.	Be able to identify their vulnerabilities to ADV.	Link CSA to erroneous beliefs about relationships and sex.	Feel confident in using the principles of effective and appropriate communication in their interpersonal relationships.	Be able to identify resource persons.
Self-esteem	Discuss how important it is for them to be in a healthy, equal relationship.	Enhance their ability to recognize the signs of violence in relationships.	Discuss the importance in terms of asserting one’s self in the face of nonconsensual sexual activity.	Discuss how important it is for them to be able to assert themselves.		
External determinants						
	The interveners are going to . . .					
Resources	Facilitate access to resources that promote healthy and equal relationships within and outside the center.	Facilitate access to resources that prevent dating violence within and outside the center.	Inform participants of the resources available outside the center.			
Social support	Reinforce participants who recognize the importance of respecting their limits in relationships	Encourage participants who recognize the signs of violence.	Encourage participants who recognize their vulnerability to revictimization.	Encourage participants to recognize erroneous beliefs related to romantic and sexual relationships.	Encourage participants to use appropriate and effective communication principles.	Encourage participants to seek resources.

*Note.* CSA = child sexual abuse; ADV = adolescent dating violence.

### Step 2: Analysis of the Conceptual Framework of the Original Program

Analysis of the conceptual framework of the program offered was carried out. To do so, we conducted a thematic analysis of the program content. The coding grid used for analyzing the conceptual framework included clientele characteristics, program objectives, practical applications, and themes addressed. The risk factors and learning objectives addressed in the program have been identified. The logic model of the initial program was derived at the end of this step. Thematic analysis was conducted on the original program and helped us to identify some risk factors and learning objectives in the matrix that were not covered. Table 3 presents the initial program logic model, the risk factors not addressed, and examples of learning objectives not met.

**Table 3:** Logic Model of the Original Program

Clientele	Objectives	Main themes	Practical applications	Program values	Risk factors not addressed in the current program	Examples of matrix learning objectives not met
Groups of 6–12 adolescent girls aged 13–17 who have experienced CSA.	• Reduce the impacts of CSA; • Develop strategies to better manage the consequences of CSA; • Help participants discover and use their personal resources and those of others around them; • Break the silence and the feeling of being alone after experiencing CSA; • Prevent revictimization.	• Cycle of abuse, the consequences of CSA; • Impact of CSA on sexuality and relationships; • Prevention of revictimization; • Communication.	• Games; • Videos; • Testimonials and discussions; • Letter writing; • Anonymous questions; • Collages; • Brainstorming; • Individual work; • Roleplaying.	• Denouncing ADV and valuing healthy and equal romantic relationships; • Animation of the group led by a meaningful adult.	• Difficulty recognizing violence and healthy, equal relationship; • Difficulty recognizing vulnerability to revictimization; • Difficulty ending an abusive relationship; • Difficulty seeking help and support when needed (including isolation); • Difficulty in assertiveness and identifying the notion of equality between two individuals; • Foundations of valid sexual consent that may be erroneous; • Loss of hope for a harmonious and equal romantic relationship.	• Define their needs within a healthy and egalitarian romantic relationship; • Be able to explain the elements of valid, free and informed sexual consent; • Identify strategies for asserting themselves in their interpersonal relationships; • Reinforce participants who list ways to overcome assertiveness challenges.

*Note.* CSA = child sexual abuse; ADV = adolescent dating violence.

### Step 3: Selection of Interventions and Developing New Interventions

In the third step, we selected the evidence-based intervention components to be adapted, modified, or developed to meet the objectives of the matrix. Interventions to be adapted were also selected based on risk factors and learning objectives not covered in the original program. To select the interventions to be adapted, a thematic analysis was carried out with the coding grid used in step 2. Then, the information was compared to the characteristics of the new clientele. It was guided by the following questions: Are the interventions adapted to the developmental stage of the clientele? Should the content of the intervention be adapted to address the specific needs of the clientele? Is the proposed intervention feasible and realistic regarding duration and adequate to the group format? In terms of context adaptation, the characteristics and needs identified in the first step must be taken into account to ensure that the intervention is adapted to the clientele. Bartholomew and colleagues (2016) also argue that theoretical methods should underpin the practical applications (activities).

Interventions adapted to the new setting are produced at the end of this step and are ready to be presented to practitioners (Bartholomew et al., 2016).

### Step 4: Validation With a Committee of Practitioners

In step 4, we formed a committee to validate the interventions through a 90-minute focus group. With their consent, the discussion was recorded and transcribed verbatim. This consultation involved seven persons including four key informants working at a center for victims of sexual assault (psychologist and social worker) as well as three members of the research team. Each intervention planned was discussed with the committee. Practitioners were invited to comment on the content, the pedagogical strategies, and the relevance of the interventions for the priority population. They were also invited to provide recommendations to improve the final version of the interventions. Following the meeting with the committee, their comments were summarized and modifications were applied by the research team in light of the comments received. Owing to the sequence of the program and the fact that at the time of implementing the new interventions, the youth were at the end of the therapy, it was not possible to obtain feedback from youth regarding all interventions planned. Youth provided comments on one intervention regarding the content, pedagogical methods, and suggestions for improvement to the practitioner, who then forwarded these comments to the research team for further bonification of the intervention. Finally, the revised version of the interventions were sent back for final adjustments and were approved by the practitioners.

#### Ethical Considerations

This study received the approval of the institutional research ethics board of the authors’ affiliated University.

## Results

Outcomes of the methodology employed will be described according to the following four steps.

### Step 1: Needs Assessment

Scientific literature clearly suggests that adolescent girls who have experienced CSA are at risk of revictimization; more specifically, this revictimization lies in the form of psychological, physical, or sexual victimization in the context of dating relationships when compared to girls who have not experienced CSA. Furthermore, key informants confirmed that revictimization was a significant issue in the clientele seen in the community setting. It was proposed to add interventions about the impact of CSA on sexuality and sexual consent, in addition to issues surrounding romantic relationships and to target assertiveness, setting limits, and ways to ensure these are respected. Informants reported that planned activities were perceived as interesting, ludic, and useful by the adolescent girls, both in the context of viewing video materials and group discussions.

A total of 21 risk factors were found from literature review and consultation with key informants. Figure 1 outlines the main risk factors associated with revictimization.

Since the ultimate objective of the program is to develop “appropriate strategies to reduce revictimization factors,” we had to identify performance objectives and determinants (personal and external) that could meet the objective. The personal and external determinants were selected on the basis of their proven effect in reducing revictimization risk. See Table 2 for the matrix of change summary.

### Step 2: Analysis of the Conceptual Framework of the Original Program

An overview of the original program is presented in Table 3. Of the 21 risk factors identified in the needs assessment, 14 were addressed in the original program and seven were not. In addition, some of the learning objectives from the matrix were not met (20/70). Therefore, it is necessary to consider these gaps.

### Step 3: Selection of Interventions and Developing New Interventions

Following the needs assessment and the analysis of the conceptual foundations of the original program, two interventions derived from universal prevention programs have been adapted, while two new interventions were developed. The two interventions that have been adapted will first be described as well as their relevance to the new clientele. Next, the interventions that have been created will be presented. Table 4 presents an overview of interventions objectives, practical application, and risk revictimization factors addressed.

**Table 4:** Interventions Summary

Intervention	Objectives	Practical application	Risk factor associated to revictimization
A Relationship in My Image	• Reflecting on relationships; • Reflecting on one’s own representations of love relationships; • Becoming aware of aspects to be promoted for healthy romantic relationships; • Identifying the important components of a healthy romantic relationship that are important to you; • Acknowledging that it is possible to have a healthy romantic relationship despite the sexual assault experienced.	• Discussion; • Individual reflection; • Plenary session.	• Difficulty distinguishing an abusive relationship from a healthy relationship; • Mistaken beliefs related to sexuality and relationships; • Loss of hope about the future (including having an healthy romantic relationship).
Julia’s Blog	Learning to identify the signs of an abusive relationship; • Pointing out certain myths conveyed in the context of a romantic relationship; • Knowing actions that can be taken to support a person who is experiencing DV; • Identifying trusted people or accessible resources that can provide support when needed; • Knowing some of the risk factors associated with revictimization.	• Teamwork; • Plenary session; • Discussion.	• Difficulty distinguishing an abusive relationship from a healthy relationship; • Mistaken beliefs related to sexuality and relationships; • Difficulty ending an abusive relationship; • Feeling of powerlessness; • Difficulty recognizing their vulnerability to revictimization; • Difficulty seeking help when necessary.
Sexual Consent	• Knowing the elements of valid, free, and informed sexual consent; • Identifying ways to make it easier to assert one’s self to resist non-consensual sexual activity	• Video presentation; • Discussion • Team game; • Plenary session.	• Mistaken beliefs related to sexuality and relationships; • Assertiveness and communication difficulties.
My Priority? Equality!	• Recognize their level of comfort with assertiveness in their interpersonal relationships.• Determine their difficulties in asserting themselves in their interpersonal relationships.	• Brainstorming; • Game; • Plenary session.	• Assertiveness and communication difficulties • Feeling of powerlessness.

### Adapted Interventions

#### Intervention 1: A Relationship in My Image

“A Relationship in My Image” is an adapted version of the “Seven Words and a Picture to Promote Healthy Relationships,” developed as part of the knowledge transfer campaign of the Youth Romantic Relationship (YRR) project (Lavoie et al., 2015). The intervention was developed based on the results of a representative study of youth (*n* = 6531) from Quebec province documenting their romantic relationship pathways and translated the results in user-friendly interventions for teachers or support staff. This intervention is appropriate for CSA victims in terms of age, gender, or developmental stage, the relevant themes, and the objectives pertinence to meet priority population needs. In this intervention, participants reflect on the components of a healthy and egalitarian romantic relationship using words named by adolescent girls who have experienced CSA. Misconceptions about romantic relationships and the impact of CSA on ADV are also addressed. Finally, they identify five elements that they wish to find and are important to them in a romantic relationship.

#### Intervention 2: Julia’s Blog

“Julia’s Blog” is an intervention derived from the *Teens Promoting Equality and Preventing Violence in Teen Dating Relationships* (ViRAJ) program (Lavoie et al., 2009). This program was evaluated and found to be effective in increasing knowledge about and increasing attitudes toward ADV (Lavoie et al., 2011). The intervention adapted is the additional intervention “Letters or emails.” This intervention was chosen to address ADV and help-seeking. The practical applications and content offered are consistent with CSA clientele topics (i.e., healthy romantic relationships, dating violence in romantic relationships, including control and jealousy). Moreover, given both the similarities regarding the ViRAJ program and the common values underlying it (e.g., valuing healthy and egalitarian romantic and interpersonal relationships, clearly attributing responsibility to actions of the perpetrator), combined with objectives pursued by the community setting, this intervention was considered a relevant choice for adaptation. Through a fictitious blog article, participants are asked to identify the signs of an abusive romantic relationship as well as to deconstruct myths maintained. In addition, they discuss the importance of identifying people they can trust or available resources if they wish to obtain support.

### Developed Interventions

#### Intervention 3: Sexual Consent

This intervention was planned to raise awareness about free and informed sexual consent. Using a public access humorous video (Tea Consent; Blue Seat Studio, 2015) and a team quiz game, participants are invited to reflect on sexual consent and integrate new notions related to this topic. This intervention allows participants to learn the key elements of valid sexual consent and discuss facilitators and barriers to voicing consent. By inviting young people to reflect on sexual consent, they will be better able to recognize situations where their consent would not be free and informed.

#### Intervention 4: My Priority? Equality!

Assertiveness is a protective factor against revictimization (Cuevas et al., 2010). This intervention was developed to address assertiveness in different context (friends, family, and romantic relationships). Using a reflexive game, participants are invited to position themselves in relation to their comfort level in terms of asserting themselves in different interpersonal contexts. They are also asked to reflect on the reasons why they may be less comfortable in different contexts. By being assertive, girls can regain power over their lives and feel that they can successfully exit abusive interactions early and feel more empowered to end an abusive relationship.

### Step 4: Validation With a Committee of Practitioners

Each previously described intervention was presented to the committee. After discussing the content, practical application and relevance to the clientele, a content analysis was carried out. The final versions of the four interventions are available in French: https://martinehebert.uqam.ca/en/.

## Discussion

This study presented the systematic approach used to contextually adapt and develop interventions aiming to prevent revictimization in romantic relationships for CSA victims. The step-by-step guidance provided by IM ensures rigor and a fair identification of intervention priorities. Following four steps, this process resulted in a final version of the interventions that are implemented in the original program.

First, our analysis underscores the importance of considering the multiple causes and risk factors of a problem to develop interventions that are adapted to the needs of the priority population. Indeed, the needs assessment, which was the first step in the process, provided an in-depth understanding of the issue by identifying the behavioral and environmental factors associated with revictimization. Identifying risk factors for revictimization has been recognized in the scientific literature as a necessary first step when developing a program (Boivin et al., 2014). The targeted intervention approach is appropriate and definitely meets this recommendation, as the interventions developed are evidence-based, including risk factors, and the perspective of a committee of practitioners working closely with the priority population are considered. Interventions were designed with the intention that the participants would be able to understand and consolidate the new learning in their daily lives. According to Castro and his colleagues (2004), these criteria are essential conditions for offering relevant intervention for a new setting.

Second, the results show the benefit of working closely with the clinical team to adapt and develop interventions. This collaborative approach has had the advantage of ensuring that the interventions adapted and developed meet the needs of both the practitioners and the adolescents. In addition, during the validation process, the implementation of the proposed interventions in their current program was discussed with practitioners. This made it possible to discuss the new sequence of the program. As the practitioners are in agreement with the location of the proposed interventions in their program, the implementation potential is higher. All of the interventions that have been developed as part of this process are integrated into the group intervention program. Using a rigorous, iterative, and collaborative approach based on IM, the interventions are likely to be relevant to the priority population (Bartholomew et al., 2016). Several authors support the importance of obtaining feedback from the clientele by testing the program and then collecting their comments or surveying community practitioners (Bartholomew et al., 2016; Foshee et al., 2015).

### Limits

Bartholomew et al. (2016), suggest forming a planning committee, made up of community practitioners and the priority population, to support the program adaptation process at each step. Given the constraints encountered along the process (employee turnover, limited financial resources, small team), it was not possible to set up such a committee. Instead, the research team and practitioners met at key moments in the process to validate the interventions and improve them. Also, teenage girls gave their opinion on “My Priority? Equality!” because it was the only intervention they had the opportunity to experience. At the time of implementation of the new interventions to the original program, they were at the end of a group and the sequence of the program did not allow us to test all interventions due to the sequence of the program. Although it was not possible to form an advisory committee at the beginning of the process, the interventions were revised throughout the process and with input from experts in the field of ADV and CSA. In addition, a researcher who was not part of the research team was solicited, and three experienced researchers contributed to the planinng of the interventions. Combined with a rigorous need analysis, we are convinced that the interventions developed have the potential to meet the needs of adolescent girls with a history of CSA.

In context adaptation, it is important that the theoretical methods underlying the adapted interventions are consistent with the original methods, even after adaptation. In the present context, the underlying theoretical models were not explicit in the original version of the interventions, as is often the case (Bartholomew et al., 2016). However, through a thematic analysis of these interventions, it was possible to derive both their fundamental components and a logic model. This reassures us about the that the adapted interventions remain faithful. Also, the proposed changes were supported by the evidence.

The next steps in the process, that is, the implementation and evaluation of the interventions, could be completed at a later date. To date, the effects of the interventions have not been tested, and it is therefore impossible for the moment to assess their impact on decreasing the risk of revictimization. However, the approach used fostered the elaboration of relevant material that will be used by the setting because this approach is based on evidence and the interventions have been validated and revised by several experts and a committee.

#### Implications for Practice and/or Policy and Research

The results obtained support the relevance of designing and contextually adapting interventions according to a systematic method for a priority population, leading to thoughtful decisions being made at each of the steps taken. In addition, obtaining feedback from the implementation setting when it comes to developing and implementing interventions has proven to be enriching. With the feedback from practitioners, it was possible to improve the interventions to ensure that they corresponded to the needs and characteristics of the clientele and the community setting. Other research teams may be inspired by this approach to adapt and design interventions for other public health problems.
